# Conditions of employment, work and quality of life of men and women in informal jobs

**DOI:** 10.11606/s1518-8787.2022056003505

**Published:** 2022-03-29

**Authors:** Álvaro Besoain-Saldaña, Gustavo Agurto Flores, Tiare Alarcón Muñoz, Jame Rebolledo Sanhueza

**Affiliations:** I Universidad de Chile Facultad de Medicina Departamento de Kinesiología Santiago Chile Universidad de Chile. Facultad de Medicina. Departamento de Kinesiología. Santiago, Chile; II Universidad de Chile Facultad de Medicina Escuela de Kinesiología Santiago Chile Universidad de Chile. Facultad de Medicina. Escuela de Kinesiología. Santiago, Chile; III Universidad de Chile Facultad de Medicina Núcleo Desarrollo Inclusivo Santiago Chile Universidad de Chile. Facultad de Medicina. Núcleo Desarrollo Inclusivo. Santiago, Chile

**Keywords:** Gender Inequality, Working Conditions, Employment, Informal Sector, Quality of Life

## Abstract

**OBJECTIVE:**

Identify gender gaps in the employment conditions, work and quality of life of informal sellers in Vega Central of Chile.

**METHODS:**

We conducted a cross-sectional study with eighty workers, who answered modules of the *Encuesta Nacional de Condiciones de Empleo, Trabajo y Salud* (ENETS – National Survey of Employment, Work and Health Conditions) and the SF-36 Health Questionnaire for data collection. We performed a descriptive analysis to determine the characteristics of the population and the Chi-square test to study correlations between each of the variables with gender.

**RESULTS:**

Of the sample, fifty (62.5%) are male and thirty (37.5%) are female. Both groups have similar conditions of employment, work and quality of life. However, women express greater fear than men do with respect to demanding better working conditions, as well as more discouragement and sadness during the workday and a worse perception of their state of health.

**CONCLUSIONS:**

Strategies for the promotion and prevention of occupational health and social security should consider a gender perspective on working conditions and health indicators, allowing women to develop tools to demand fair conditions and promote employer obligations to care for the well-being of male and female workers.

## INTRODUCTION

*Vega Central* is an urban market with high concentration of commercial activity in the Metropolitan Region of Chile. There are two main jobs: goods carriers, who transport and deposit goods in warehouses; and kiosk and market stall sellers, who buy and sell food products^1^. In total, Vega Central includes around 1,200 stores^a^.

These labor activities are mostly carried out in informal conditions, understood as those jobs that have little or no social protection, are not subject to labor legislation, have no right to benefits and are not subject to taxation^2^. In Latin America and the Caribbean, at least 50% of workers work informally^3^ and, of all women who work, 59% are in informal jobs^4^.

In this context, gender is a social construct that produces health inequities on its own, which can be accentuated if it interacts with other social determinants of health^5^ such as occupation and working conditions. Worldwide, women are more likely to have poorer working conditions than men, such as greater difficulty in covering their expenses with wages, longer working hours, more hours of involuntary part-time work, less training and health and safety information about their workplace, fewer non-wage benefits, and less communication with their superiors^6^.

Informal employment presents conditions of high vulnerability, especially in the face of risk situations^2^. There is a relationship between informal work and poorer health^7,8^, with the self-perceived health of informal workers being worse than that of formal workers^9^. At the same time, informal workers are exposed to greater occupational accidents; they do not have coverage for health care, unemployment insurance or retirement^10^. In contrast, there is literature that found no evidence on the relationship between informal work and poorer health^6^.

While most studies suggest that the results are more unfavorable for the female population^8,9,11^one study reported worse effects of informality in men with respect to self-perceived and mental health^7^. In the Chilean context, the *Encuesta Nacional de Condiciones de Empleo, Trabajo y Salud* (ENETS - National Survey of Employment, Labor and Health Conditions), published in 2011^14^, shows that labor informality has a negative impact on the perception of mental or emotional health, affecting mostly women. However, the information on informal employment in Chile and its impact on workers’ health are scarce, does not specify the categories analyzed and the differences by gender are still unclear.

With this in mind, this study aims to identify gender gaps in the employment conditions, work and quality of life of informal sellers in Vega Central, in Chile. Thus, the expectation is that, under the same conditions of employment and work in this area, women with informal labor have worse health and quality of life outcomes than men have. This study provides background information for the development of prevention and promotion of occupational health from a gender perspective.

## METHODS

### Study Design and Participants

We performed a quantitative, non-experimental, cross-sectional descriptive study. The representative population was 317 stalls selling vegetables and fruits in Vega Central of Santiago, Chile. We estimated a sample size^15^ based on the proportion of informal work at the national level^14^, given the lack of knowledge of the total number of informal workers in Vega Central. We obtained an estimated sample size of 148 subjects, with a confidence interval of 95% and a precision of 3%. We reached a final sample of 80 workers without a current employment contract, who had been involved in sales activity for at least 3 months and who communicated in Spanish (Figure). Given the existence of a high degree of heterogeneity in the working and living conditions of this sector^16^, we performed a post hoc analysis of the statistical power of the sample in order to reduce type II errors in the interpretation.

**Figure:**
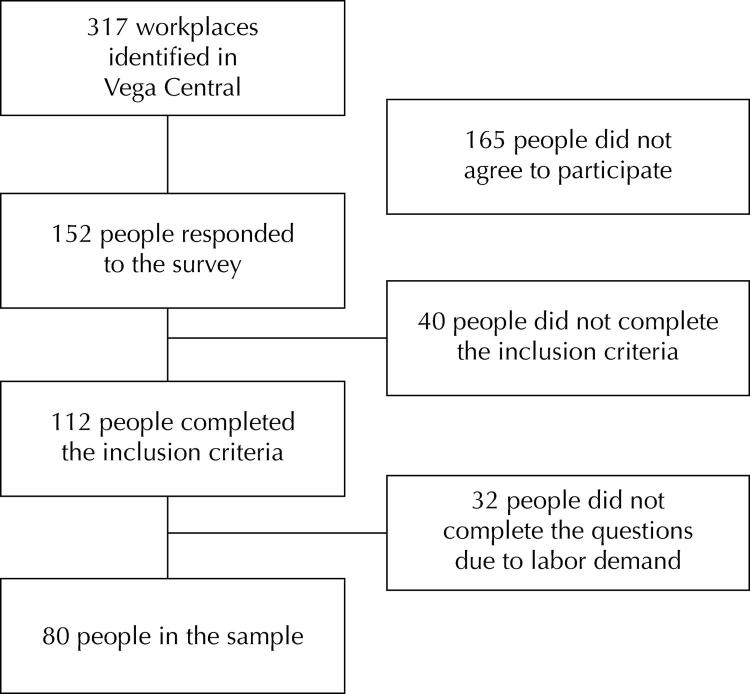
Flowchart of the study sample.

### Data Collection and Variables

A research team, trained during the second half of 2018, collected data, based on a structured survey on socio demographic data (age, gender, commune of residence and educational level), employment conditions, working conditions and quality of life. We considered the gender category in a binary dimension for statistical power criteria of each group. We used this variable for gender analysis by comparing roles and working conditions.

On the other hand, we used modules A and B of the ENETS^14^ to measure employment and work conditions, while the quality of life was measured using the SF-36^17^ questionnaire. For the application of the instruments, we previously read the complete set of questions and answer options. We obtained weight and height numbers of each worker by self-reporting.

### Data Analysis

We described frequencies and percentages for the analysis of qualitative variables. Subsequently, we used the chi-square test to analyze the associations between the variables: gender – employment conditions; gender – working conditions; and gender – quality of life. We considered p value ≤ 0.05 and Haberman^18^ adjusted standardized residuals as ≤ -1.96 and ≥ 1.96 to identify significant associations. We used the statistical program IBM SPSS Statistics for Macintosh, version 22.0 (IBM Corporation, Armonk, NY).

The Human Research Ethics Committee of the *Facultad de Medicina de la Universidad de Chile* approved this study, assigned no. 80, dated September 12, 2017.

## RESULTS

Of 80 people surveyed, 62.5% were male and 37.5% were female, corresponding to 50 and 30 people, respectively. The median age of the women surveyed was 44 years, while the median age of the men was 50 years. Table 1 shows the sociodemographic characteristics of the participants.

**Table 1:** Sociodemographic characterization of workers in Vega Central, Chile.

Variables		Gender	

		Female (%) n = 30	Male (%) n = 50
Age (years)^a^		44 (16)	50 (26)
Weight (kg)^a^		68 (9)	81 (13)
Height (m)^a^		1.59 (0.08)	1.72 (0.08)
BMI (kg/m^2^)^a^		27.0 (2.6)	27.7 (3.9)
Educational level (attending or completed) Count (%)	Never attended	0 (0.0)	1 (2.0)
	Elementary	12 (40.0)	15 (30.0)
	High School	14 (46.7)	23 (46.0)
	Technical/superior	4 (13.3)	11 (22.0)
Type of contract Count (%)	Verbal	5 (16.7)	12 (24.0)
	Does not have	25 (83.3)	38 (76.0)
Type of work Count (%)	Permanent	24 (80.0)	44 (88.0)
	Temporary	6 (20.0)	6 (12.0)
Time on current job Count (%)	Less than 1 year	6 (20.0)	8 (16.0)
	Between 1 year and 10 years	14 (46.7)	15 (30.0)
	More than 10 years	10 (33.3)	27 (54.0)

^a^ Variables described with median and interquartile range, 75 percentile (p75) - 25 percentile (p25).

With respect to employment conditions, only three variables showed significant differences in relation to gender (Table 2). Men were at a disadvantage in the number of Sundays worked (p = 0.011) and regarding the need to make up for unforeseen expenses (p = 0.033). Thus, 80.6% of men reported having worked three or all Sundays per month and 66.6% had unexpected expenses to pay, unlike 56.7% and 40% of women, respectively. In contrast, the results for the variable “fear of demanding better conditions” were worse for women (p = 0.002). Women were 23.3% “almost always or always” afraid of demanding better employment conditions, unlike 2% of men.

**Table 2:** Characterization of the variables of the workers of Vega Central (Chile) by gender.

Variables		Gender		p

		Female Count (%) [adjusted residual]	Male Count (%) [adjusted residual]	
Sundays worked	No Sunday/one Sunday per month	8 (26.6) [-2.5]	4 (8.0) [2.5]	0.011
	Three Sundays a month/every Sunday	17 (56.7) [2.5]	43 (86.0) [-2.5]	
Supplements unforeseen expenses	Never/rarely	17 (56.7) [2.1]	17 (34.0) [-2.1]	0.033
	Almost always/always	12 (40.0) [-2.1]	33 (66.0) [2.1]	
Fear of claiming better conditions	Never/rarely	22 (73.4) [3.1]	48 (96.0) [-3.1]	0.002
	Almost always/always	7 (23.3) [-3.1]	1 (2.0) [3.1]	
Helpless in the face of superiors	Never/rarely	26 (86.7) [1.6]	47 (94.0) [-1.6]	0.113
	Almost always/always	3 (10.0) [-1.6]	1 (2.0) [1.6]	
Fear of dismissal	Never/rarely	24 (80.0) [1.6]	46 (92.0) [-1.6]	0.118
	Almost always/always	5 (16.6) [-1.6]	3 (6.0) [1.6]	
Unfair treatment	Never/rarely	27 (90.0) [0.1]	46 (92.0) [-0.1]	0.893
	Almost always/always	2 (6.7) [-0.1]	3 (6.0) [0.1]	
Violent treatment	Never/rarely	28 (93.3) [1.3]	49 (98) [-1.3]	0.191
	Almost always/always	1 (3.3) [1.3]	0 (0.0) [1.3]	
Use of sick leave	Never/rarely	22 (73.3) [-0.5]	34 (68.0) [0.5]	0.614
	Almost always/always	8 (26.7) [0.5]	16 (32.0) [-0.5]	
Social security system	Yes	4 (13.3) [1.2]	12 (24) [-1.2]	0.113
	No	20 (66.7) [0.3]	35 (70) [-0.3]	
	Does not know	6 (20) [-1.9]	3 (6) [-1.9]	

Women and men work under the same conditions in most cases (Table 3). Two variables showed significant differences in relation to gender. Women presented worse results in terms of exposure to low temperatures (p = 0.012), with 76.7% being exposed during the whole day or half of it, while men reported 48%. Regarding whether the company was concerned about their health and safety, there was a high degree of heterogeneity in results, so we did not observe a clear trend despite finding a significant association (p = 0.034). We identified specific differences between 33.3% of women and 10% of men, who stated that “sometimes” the company was concerned about their health and safety (adjusted standardized residual: -2.5).

**Table 3:** Characterization of working conditions and employment of workers in Vega Central (Chile) by gender.

Variables		Gender		p

		Female Count (%) [adjusted residual]	Male Count (%) [adjusted residual]	
Noise	All working day/ half working day	12 (40.0) [0.0]	20 (40.0) [0.0]	1.000
	Occasionally/never	18 (60.0) [0.0]	30 (60.0) [0.0]	
High temperatures	All working day/ half working day	10 (33.4) [-0.5]	14 (28.0) [0.5]	0.614
	Occasionally/never	20 (66.6) [0.5]	36 (72.0) [-0.5]	
Low temperatures	All working day/ half working day	23 (76.7) [-2.5]	24 (48.0) [2.5]	0.012
	Occasionally/never	7 (23.3) [2.5]	26 (52.0) [-2.5]	
Light	All working day/ half working day	4 (13.3) [1.2]	12 (24.0) [-1.2]	0.240
	Occasionally/never	25 (83.4) [-1.2]	36 (72.0) [1.2]	
Sunlight	All working day/ half working day	3 (10.0) [0.2]	6 (12.0) [-0.2]	0.816
	Occasionally/never	25 (83.4) [-0.2]	42 (84) [0.2]	
Awkward postures	All working day/ half working day	16 (53.3) [-0.9]	21 (42.0) [0.9]	0.365
	Occasionally/never	14 (46.7) [0.9]	28 (56.0) [-0.9]	
Carrying heavy objects	All working day/ half working day	13 (43.3) [1.1]	28(56.0) [-1.1]	0.273
	Occasionally/never	17 (56.7) [-1.1]	22 (44.0) [1.1]	
On straining the voice	All working day/ half working day	5 (15.7) [0.0]	9 (18.0) [0.0]	0.987
	Occasionally/never	22 (73.3) [0.0]	40 (70.0) [0.0]	
Works sitting	All working day/ half working day	11 (36.7) [-1.7]	10 (20.0) [1.7]	0.092
	Occasionally/never	18 (60.0) [1.7]	39 (78.0) [-1.7]	
Works standing	All working day/ half working day	26 (86.7) [1.1]	47 (94.0) [-1.1]	0.261
	Occasionally/never	4 (13.3) [-1.1]	3 (6.0) [1.1]	
Use of gloves	Yes	12 (40.0) [-0.9]	15 (30.0) [0.9]	0.360
	No	18 (60.0) [0.9]	35 (70.0) [-0.9]	
Use of sunscreen	Yes	2 (6.7) [-1.1]	1 (2.0) [1.1]	0.287
	No	28 (93.3) [1.1]	49 (98.0) [-1.1]	
Probability of accident	Very likely	11 (36.7) [-0.4]	16 (32.0) [0.4]	0.894
	Unlikely	15 (50.0) [0.2]	26 (52.0) [-0.2]	
	Not likely	4 (13.3) [0.3]	8 (16.0) [-0.3]	
The company cares about your health and safety	Always/almost always	9 (30.0) [1.5]	23 (46.0) [-1.5]	0.034
	Sometimes	10 (33.3) [-2.5]	5 (10.0) [2.5]	
	Rarely/never	11 (36.7) [0.5]	21 (42.0) [-0.5]	

We observed some differences by gender with respect to quality of life (Table 4), the variable “felt discouraged and sad” presented significant differences (p = 0.014), whose results were unfavorable for women, 53.3% of whom perceived that almost all or sometimes they felt discouraged or sad, as opposed to 26% of the male population. Additionally, in contrast to 12% of men, 33.3% of women rated the statement “I feel that my health is excellent” as “almost always false or definitely false” (adjusted standardized residual: -2.3).

**Table 4:** Characterization of health status and quality of life of workers in Vega Central (Chile) by gender.

Variables		Gender		p

		Female Count (%) [adjusted residual]	Male Count (%) [adjusted residual]	
Difficulties in performing work/activity	Most of the time/sometimes	11 (36.7) [-0.2]	17 (34.0) [0.2]	0.809
	Rarely/never	19 (63.3) [0.2]	33 (66.0) [-0.2]	
Doing less than desired due to emotional problems	Most of the time/sometimes	8 (26.7) [-1.0]	9 (18.0) [1.0]	0.318
	Rarely/never	21 (70.0) [1.0]	41 (82.0) [-1.0]	
Felt discouraged and sad	Almost all the time/sometimes	16 (53.3) [-2.5]	13 (26.0) [2.5]	0.014
	Very little time/never	14 (46.7) [2.5]	37 (74.0) [-2.5]	
Felt exhausted	Always/almost all the time	25 (83.3) [-0.9]	36 (72) [0.9]	0.386
	A little/very little time	5 (16.7) [-0.9]	12 (24) [-0.9]	
I feel that I get sick more easily	Definitely true/ almost always true	5 (16.7) [-1.9]	2 (4.0) [1.9]	0.057
	Almost always false/definitely false	24 (80.0) [1.9]	45 (90.0) [-1.9]	
I feel I am as healthy as anyone else	Definitely true/ almost always true	22 (73.3) [1.3]	45 (90.0) [-1.3]	0.200
	Almost always false/definitely false	4 (13.3) [-1.3]	3 (6.0) [1.3]	
I feel my health is excellent	Definitely true/ almost always true	16 (53.3) [1.9]	37 (74.0) [-1.9]	0.065
	I don’t know	4 (13.3) [0.1]	7 (14.0) [-0.1]	
	Almost always false/definitely false	10 (33.3) [-2.3]	6 (12.0) [2.3]	

Regarding the identification of such self-perception, we developed correlations with respect to quality of life variables, recognizing common associations in both genders between self-perception of health and limitation in walking more than 10 blocks (-0.417); limitation when climbing only one floor (-0.477); limitation of activities due to intense efforts (-0.412); limitation when walking several blocks (-0.390); limitation when climbing several floors (-0.567). On the other hand, we identified correlations only for women, such as limitation to moderate and minimal effort (-0.375 and -0.393, respectively), while in men, significant negative correlations were obtained in the variables “I feel that I get sick more easily” (-0.288); limitations in work/activity (-0.296); comparison of health status with the previous year (-0.299); reduction of work time due to emotional problems (-0.344); limitation when walking only one block (-0.334); limitation when bathing or dressing (-0.354); felt discouraged and sad (-0.354); limitation when squatting (-0.366) and limitation when lifting something light (-0.369).

## DISCUSSION

This study sought to identify gender differences in the employment conditions, work and quality of life of informal workers in Vega Central. It did not identify major gender differences in employment and working conditions, however women express a greater fear than men do with demanding better conditions, as well as feeling more discouraged and sad during the workday.

Compared with national data from the same survey used as a basis (ENETS, 2011), it is evident that at the national level 10.7% of men and 3.7% of women who are informal workers do not have a social security system, while in Vega Central over 60% of both genders report not being affiliated to a social security system, even when the public health system has affiliation for people with no formal income. On the other hand, 20% of women workers in this market do not know if they have health insurance, demonstrating greater vulnerability in this group.

Although the female and male population in the workplaces studied do not feel defenseless before their superiors, do not fear being dismissed, and do not perceive unfair or violent treatment, women are more fearful than men of demanding better working conditions. Considering that the global trend of informal employment describes that women receive lower remuneration for their work and at the same time are exposed to greater decent work deficits^19^, it is important in this context to implement social security strategies with a gender perspective allowing women to develop tools and spaces to demand fair conditions.

Most of the participants do not have a formal social security system, not presenting gender gaps, which is contrary to what has been reported in other studies on informal work^12^, in which the lack of social security is higher among women than among men (4% versus 1.5%). It is possible to attribute this lack of significant difference to the fact that the population analyzed is working in a not feminized or masculinized job, as other jobs in the informal sector might be. Because of the health impact this entails, it is a factor to consider knowing that, in developing countries, the informal sector is the main source of employment for women^4^.

While, in men’s and women’s perception, there is heterogeneity of the employer’s concern about their health and safety, we identify a trend towards greater perception of neglect by women (p = 0.034). We could link this difference to cultural aspects regarding the greater relevance given to health and safety in the female role. However, such negligence is present in similar reports of low access to training, information and personal protection elements in their work. This also shows that in this workplaces no clear practices exist with respect to the employer’s obligations to care for the welfare of workers, which is consistent with other studies on informal labor^6,12,19^.

In general, we identified only physical factors (awkward postures, carrying heavy objects, working days standing up) that presented exposure in more than half of the sample, while overall exposure levels of under 30% to noise, high and low temperatures or light during the working day. The only difference between genders in working conditions was identified in the high level of exposure of women to low temperatures (p = 0.012), which could be attributed to the fact that most of the day women report that they are seated and engaged in selling in an open space with a high ceiling that allows air circulation, such as the shed where Vega Central stalls are located. In contrast, similar international and national studies identified that men are more exposed to work in awkward postures^12^, under high physical demands and suffer more injuries from work-related accidents^20^, along with higher levels of work standing up, repetitive movements, lifting or carrying heavy objects, among others^14^. Despite the way in which female and male identities are configured^21^ to determine the distribution of women and men in the labor market, in general, the above results reflect an unclear division of tasks or working conditions by gender, being both equally demanded.

In relation to quality of life, although most of the workers felt exhausted most of the time, they did not report difficulties in performing their work activities or doing less than desired due to emotional problems. However, women perceive themselves to be discouraged and sad during the workday more than 2.01 (95%CI: 1.16–3.50) times than men, which can be attributed to several factors, among them, the different openness to recognize the mood of men and women, since according to the hegemonic mandate of masculinity a man should not be weak or recognize sadness. On the other hand, this finding is consistent with statistics on mental health in the general population, since women have a higher prevalence of depressive symptoms than men^22^. In addition, the literature on social determinants of health and its impact on the social gradient of health places mental health problems and disorders with a higher prevalence in population groups with lower educational levels and younger age^23,24^, an element to consider given the low educational level presented in the sample in general and in women compared to men. However, another study on the context of informality in Central America^11^ reports that both men and women have a poor perception of mental health. These results are similar to those reported in Chile, where unstable and informal workers have significantly lower levels of mental wellbeing than stable and formal workers^14^, so that discouragement and sadness could be equally associated with or enhanced by informal work and low educational level, which is also one of the reasons for accessing this type of employment.

Regarding self-perceived health in general, more than 60% say that is “definitely true/almost always true” that their health is excellent, 53.3% of women and 74% of men respectively. This overall similarity in self-perceived health, although not consistent with that reported in other studies^8,9,11^may be explained by the similarity of the demanding working and employment conditions in which both genders perform their jobs. In turn, when describing the categories “almost always false/definitely false”, a gap of more than double was identified between women (33.3%) and men (12.2%), with a relative risk of 2.07 (95%CI: 1.18-3.62). This association shows a greater correlation in the case of men to factors of physical and psychological origin (correlation levels close to -0.4), while in women it is mainly associated with physical factors in the tasks they perform (with significant correlations close to 0.4). Other studies explain this gap by physical and sensory risk factors, dual presence, low quality of leadership, low social support and little opportunity for skills development at work^26^ and time conflicts to conform work and family demands in women^27^.

Based on the literature, another factor that influences the self-perception of health of informal workers is the characteristics and experiences of the health systems in which the people working in Vega Central participate, such as problems in the administration of health services, bad treatment of users, and the lack of resources for the health sector^28^. Furthermore, Alfers et al.^8^ showed that in the context of labor informality in South Africa exists a strong association between low income and poorer health status, even greater than with labor formality. In other associations with health, it has been shown that exposure to psychosocial risks is also mediated by the social class to which the person belongs^28^.

The growth of the informal labor market in Latin America is recognized as a consequence of the development of the economic model, to which important attention should be paid given the trend towards informality as a result of the migratory processes of the population towards large cities^29^ , considering that migration in Chile has been increasing steadily and that health and social security policies continue to leave out of their reforms the situation of informality and the effects on the welfare of workers^30^.

Among the limitations of this study are the barriers presented in the process of recruiting participants and collecting data in the context of informal employment, both due to the time burden of the work and the extent of the instruments. Although this affects the statistical power of the data, we performed a post hoc analysis, finding values higher than 0.8 in the following variables that present gender differences: “self-perception of health”, “perception of encouragement and fear to demand better conditions”, and “concern of the companies about your health and safety at work”.

Another limitation of this study is that, given its descriptive scope, it does not identify the underlying causes of gender differences in the perception of health, so it is important to do new prospective or qualitative studies to deepen the understanding of informal work in markets or street markets.

The results of this study and its analysis allow us to conclude that, despite the fact that women and men have similar working and labor conditions in the area studied, women have a worse perception of their mental health and general health. We only identified gender differences in the perception of the employer’s concern for health and safety at work. Therefore, occupational health promotion and prevention strategies in the different areas of informal work, as well as future studies in this field, should be designed from a gender perspective, incorporating both working conditions and health and quality of life indicators. On the other hand, as employment formalization and social security policies advance, alternative strategies can be implemented to support mothers and fathers with the care of young children, such as the development of agreements with local public services to have benefits such as access to kindergartens, extended school hours programs, or daycare facilities on the same place they work. Therefore, all measurement and research on informal work requires consideration of the particularities of women that influence their performance and overall health at work.

## Footnotes

a Vegacentral.CL. Santiago (CL): Vega Central; c2020 [cited 2020 Jun 1]. Available from: https://www.vegacentral.cl/
