# Supplementary material for: Conditions of employment, work and quality of life of men and women in informal jobs
Source: Rev Saude Publica. 2022 Mar 29;56:17. doi: 10.11606/s1518-8787.2022056003505 (PMC8973026; doi:10.11606/s1518-8787.2022056003505)
Supplement: Translation

# Translation: Condiciones de empleo, trabajo y calidad de vida de hombres y mujeres en trabajo informal

**Authors:** Álvaro Besoain-Saldaña, Gustavo Agurto Flores, Tiare Alarcón Muñoz, Jame Rebolledo Sanhueza

**Affiliations:** I Chile Universidad de Chile. Facultad de Medicina. Departamento de Kinesiología. Santiago, Chile; II Chile Universidad de Chile. Facultad de Medicina. Escuela de Kinesiología. Santiago, Chile; III Chile Universidad de Chile. Facultad de Medicina. Núcleo Desarrollo Inclusivo. Santiago, Chile

**Keywords:** Inequidad de Género, Condiciones de Trabajo, Empleo, Sector Informal, Calidad de Vida

## Abstract

**OBJETIVO:**

Identificar brechas de género en las condiciones de empleo, trabajo y calidad de vida de vendedores informales de la Vega Central de Chile.

**MÉTODOS:**

Se realizó un estudio transversal con ochenta trabajadores, quienes respondieron módulos de la Encuesta Nacional de Condiciones de Empleo, Trabajo y Salud (ENETS) y al Cuestionario de Salud SF-36 para la recolección de datos. Se hizo un análisis descriptivo para determinar características de la población y la prueba de Chi-cuadrado para estudiar correlaciones entre cada una de las variables con el género.

**RESULTADOS:**

De la muestra, cincuenta (62,5%) pertenecen al género masculino, y treinta (37,5%) al género femenino. Ambos grupos presentan condiciones de empleo, trabajo y calidad de vida similares. No obstante, las mujeres manifiestan mayor temor que los hombres a reclamar mejores condiciones de trabajo, así como más desánimo y tristeza durante la jornada y peor percepción de estado de salud.

**CONCLUSIONES:**

Las estrategias de promoción y prevención de salud laboral y seguridad social deben considerar una perspectiva de género sobre las condiciones laborales e indicadores de salud, que permitan a mujeres desarrollar herramientas para demandar justas condiciones y promuevan obligaciones al empleador de preocuparse del bienestar de trabajadores y trabajadoras.

## INTRODUCCIÓN

La Vega Central es un mercado urbano que concentra una alta actividad comercial en la Región Metropolitana de Chile. Se desempeñan principalmente dos puestos de trabajos: peón de carga, quienes transportan y depositan mercancías en almacenes, y los/as vendedores/as de quioscos y puestos de mercado, quienes realizan las tareas de comprar y vender productos alimenticios^1^. En total, la Vega Central reúne cerca de 1.200 locales^a^.

Estas actividades laborales se realizan mayormente en condiciones de informalidad, entendida como aquellos empleos que tienen escasa o nula protección social, no están sujetos a legislación laboral, no poseen derecho a prestaciones y están al margen de pago de impuestos^2^. Cabe destacar que en América Latina y el Caribe, al menos el 50% de los/as trabajadores/as lo hace en condiciones de informalidad^3^ y, del total de mujeres que trabaja, el 59% lo hace en un empleo informal^4^.

En este contexto, cabe destacar que el género es una construcción social que produce inequidades sanitarias por sí sólo, las cuales pueden acentuarse si interactúa con otros determinantes sociales de la salud^5^, como la ocupación y las condiciones laborales. Mundialmente, se ha observado que existe una mayor probabilidad de precarización de las condiciones de trabajo de mujeres respecto a la de los hombres, tales como mayor dificultad para cubrir sus gastos con el salario, jornadas de trabajo más extensas, más horas de trabajo a tiempo parcial involuntario, menos capacitación e información de salud y seguridad de su lugar de trabajo, menos prestaciones no salariales y menor comunicación con sus superiores^6^.

Se ha evidenciado que el empleo informal presenta condiciones de alta vulnerabilidad, especialmente frente a situaciones de riesgo^2^. Existe una relación entre trabajo informal y peor salud^7,8^, siendo la salud autopercibida de los/as trabajadores/as informales peor que la de los/as trabajadores/as formales^9^. A su vez, están expuestos a mayores accidentes laborales, no tienen cobertura para tratamientos de salud, seguro de desempleo ni jubilación^10^. En contraste, existe literatura que no encontró evidencia sobre la relación entre trabajo informal y peor salud^6^.

Si bien la mayor parte de estudios sugieren que los resultados son más desfavorables para la población femenina^8,9,11^, un estudio ha reportado peores efectos de la informalidad en hombres respecto a la salud autopercibida y mental^7^. En el contexto chileno, la Encuesta Nacional de Condiciones de Empleo, Trabajo y Salud (ENETS), publicada el año 2011^14^, demuestra que la informalidad laboral tiene un impacto negativo en la percepción de salud mental o emocional, afectando en mayor medida a las mujeres. No obstante, la información sobre el empleo informal en Chile y sus repercusiones en la salud de los trabajadores es escasa, no precisa los rubros analizados y las diferencias por género aún no están claras.

Teniendo en cuenta esto, el presente estudio tiene por objetivo identificar brechas de género en las condiciones de empleo, trabajo y calidad de vida de vendedores informales de la Vega Central de Chile. De este modo, se espera que, bajo las mismas condiciones de empleo y trabajo en este rubro, las mujeres con informalidad laboral tengan peores resultados en salud y calidad de vida que los hombres. Este estudio permite contribuir con antecedentes al desarrollo de prevención y promoción de salud laboral desde una perspectiva de género.

## MÉTODOS

### Diseño de Estudio y Participantes

Se realizó un estudio de metodología cuantitativa, no experimental de tipo descriptivo transversal. Se consideró como población a representar 317 puestos de venta de verduras, hortalizas y frutas, de la Vega Central de Santiago, Chile. Se estimó un tamaño muestral^15^ según la proporción de trabajo informal a nivel nacional^14^, dado el desconocimiento de la cantidad total de trabajadores informales presentes en La Vega Central. Se obtuvo un tamaño muestral estimado de 148 sujetos, con un nivel de confianza del 95% y precisión del 3%. Se alcanzó una muestra final de 80 trabajadores/as sin contrato laboral vigente, que mantenían una actividad de ventas en un periodo de al menos 3 meses y se comunicaban en idioma español (Figura). Dada la existencia de una alta heterogeneidad en las condiciones de trabajo y de vida de este sector^16^, se realizó un análisis post-hoc de la potencia estadística de la muestra lograda, con tal de reducir errores tipo II en la interpretación.

**Figura:** Diagrama de flujo de la muestra del estudio.

### Recolección de Datos y Variables

La recolección de datos se realizó por parte del equipo de investigación capacitado durante el segundo semestre de 2018, a partir de una encuesta estructurada sobre datos sociodemográficos (edad, género, comuna de residencia y nivel educacional), condiciones de empleo, condiciones de trabajo y calidad de vida. La categoría de género se consideró en una dimensión binaria por criterio de potencia estadística de cada grupo. Esta variable fue utilizada para el análisis de género, mediante la comparación de roles y condiciones de trabajo.

Por otro lado, para la medición de las condiciones de empleo y trabajo se utilizaron los módulos A y B de la ENETS^14^, mientras que la medición de calidad de vida se realizó mediante el Cuestionario SF-36^17^. Para la aplicación de los instrumentos, se realizó previamente la lectura completa de preguntas y opciones de respuesta. Los valores de peso y talla de cada trabajador se obtuvieron mediante autorreporte.

### Análisis de Datos

Para el análisis de las variables cualitativas, se describieron frecuencias y porcentajes. Posteriormente, se utilizó la prueba de chi-cuadrado para analizar las asociaciones entre las variables: género – condiciones de empleo; género – condiciones de trabajo; y género – calidad de vida. Se consideró el valor de p ≤ 0,05 y los residuos tipificados corregidos de Haberman^18^ como ≤ -1,96 y ≥ 1,96 para identificar asociaciones significativas. Se utilizó el programa estadístico IBM SPSS Statistics para Macintosh, versión 22.0 (IBM Corporation, Armonk, NY).

Este estudio fue aprobado por el Comité de Ética de Investigación en Seres Humanos de la Facultad de Medicina de la Universidad de Chile, asignado al n.° 80 con fecha 12 de septiembre de 2017.

## RESULTADOS

Del total de 80 personas encuestadas, el 62,5% pertenecía al género masculino y un 37,5% al género femenino, lo que corresponde a 50 y 30 personas, respectivamente. La mediana de edad de las mujeres encuestadas fue de 44 años, mientras que la de los hombres fue de 50 años. La caracterización sociodemográfica de las personas participantes se encuentra en la Tabla 1.

**Tabla 1:** Caracterización sociodemográfica de los/las trabajadores/as de la Vega Central, Chile.

Variables		Género	

		Femenino (%) n = 30	Masculino (%) n = 50
Edad (años)^a^		44 (16)	50 (26)
Peso (kg)^a^		68 (9)	81 (13)
Talla (m)^a^		1,59 (0,08)	1,72 (0,08)
IMC (kg/m^2^)^a^		27,0 (2,6)	27,7 (3,9)
Nivel educacional (cursado o completado) Recuento (%)	Nunca asistió	0 (0,0)	1 (2,0)
	Básica	12 (40,0)	15 (30,0)
	Media	14 (46,7)	23 (46,0)
	Técnico/superior	4 (13,3)	11 (22,0)
Tipo de contrato Recuento (%)	Verbal	5 (16,7)	12 (24,0)
	No tiene	25 (83,3)	38 (76,0)
Tipo de trabajo Recuento (%)	Permanente	24 (80,0)	44 (88,0)
	De temporada	6 (20,0)	6 (12,0)
Tiempo en trabajo actual Recuento (%)	Menos de 1 año	6 (20,0)	8 (16,0)
	Entre 1 año y 10 años	14 (46,7)	15 (30,0)
	Más de 10 años	10 (33,3)	27 (54,0)

^a^ Variables descritas con mediana y rango intercuartílico, percentil 75 (p75) - percentil 25 (p25).

Respecto a las condiciones de empleo, solo tres variables presentaron diferencias significativas en relación al género (Tabla 2). Los hombres se vieron desfavorecidos en los domingos trabajados (p = 0,011) y en suplir gastos imprevistos (p = 0,033). De esta manera, el 80,6% de los hombres refirieron haber trabajado tres o todos los domingos al mes y el 66,6% tuvo que suplir gastos imprevistos, a diferencia de un 56,7% y un 40% de las mujeres, respectivamente. En contraste, los resultados de la variable “temor a reclamar mejores condiciones” fueron peores para las mujeres (p = 0,002). El 23,3% de las mujeres “casi siempre o siempre” presentó temor a reclamar mejores condiciones de empleo, a diferencia de un 2% de los hombres.

**Tabla 2:** Caracterización de las variables de los/las trabajadores/as de la Vega Central (Chile) por género.

Variables		Género		p

		Femenino Recuento (%) [residuos corregidos]	Masculino Recuento (%) [residuos corregidos]	
Domingos trabajados	Ningún domingo/un domingo al mes	8 (26,6) [-2,5]	4 (8,0) [2,5]	0,011
	Tres domingos al mes/todos los domingos	17 (56,7) [2,5]	43 (86,0) [-2,5]	
Suple gastos imprevistos	Nunca/rara vez	17 (56,7) [2,1]	17 (34,0) [-2,1]	0,033
	Casi siempre/siempre	12 (40,0) [-2,1]	33 (66,0) [2,1]	
Temor a reclamar mejores condiciones	Nunca/rara vez	22 (73,4) [3,1]	48 (96,0) [-3,1]	0,002
	Casi siempre/siempre	7 (23,3) [-3,1]	1 (2,0) [3,1]	
Indefenso ante superiores	Nunca/rara vez	26 (86,7) [1,6]	47 (94,0) [-1,6]	0,113
	Casi siempre/siempre	3 (10,0) [-1,6]	1 (2,0) [1,6]	
Temor a despido	Nunca/rara vez	24 (80,0) [1,6]	46 (92,0) [-1,6]	0,118
	Casi siempre/siempre	5 (16,6) [-1,6]	3 (6,0) [1,6]	
Tratos injustos	Nunca/rara vez	27 (90,0) [0,1]	46 (92,0) [-0,1]	0,893
	Casi siempre/siempre	2 (6,7) [-0,1]	3 (6,0) [0,1]	
Tratos violentos	Nunca/rara vez	28 (93,3) [1,3]	49 (98) [-1,3]	0,191
	Casi siempre/siempre	1 (3,3) [1,3]	0 (0,0) [1,3]	
Uso de licencias médicas	Nunca/rara vez	22 (73,3) [-0,5]	34 (68,0) [0,5]	0,614
	Casi siempre/siempre	8 (26,7) [0,5]	16 (32,0) [-0,5]	
Sistema previsional	Sí	4 (13,3) [1,2]	12 (24) [-1,2]	0,113
	No	20 (66,7) [0,3]	35 (70) [-0,3]	
	No sabe	6 (20) [-1,9]	3 (6) [-1,9]	

Se observó que mujeres y hombres trabajan bajo las mismas condiciones en la mayor parte de los casos (Tabla 3). Dos variables mostraron diferencias significativas en relación al género. Las mujeres presentaron peores resultados en cuanto a la exposición a bajas temperaturas (p = 0,012), siendo el 76,7% quienes lo hicieron durante toda la jornada o la mitad de ella, mientras que los hombres reportan un 48%. Respecto a si la empresa se preocupaba de su salud y seguridad, se presenta una alta heterogeneidad en sus resultados, por lo que no se observó una tendencia clara pese a encontrar una asociación significativa (p = 0,034). Específicamente, solo se identificaron diferencias entre el 33,3% de las mujeres y el 10% de los hombres, quienes declararon que “algunas veces” la empresa se preocupaba por su salud y seguridad (residuos tipificados corregidos: -2,5).

**Tabla 3:** Caracterización de condiciones de trabajo y empleo de trabajadores de la Vega Central (Chile) por género.

Variables		Género		p

		Femenino Recuento (%) [Residuos corregidos]	Masculino Recuento (%) [Residuos corregidos]	
Ruido	Toda la jornada/mitad de la jornada	12 (40,0) [0,0]	20 (40,0) [0,0]	1,000
	Ocasionalmente/nunca	18 (60,0) [0,0]	30 (60,0) [0,0]	
Altas temperaturas	Toda la jornada/mitad de la jornada	10 (33,4) [-0,5]	14 (28,0) [0,5]	0,614
	Ocasionalmente/nunca	20 (66,6) [0,5]	36 (72,0) [-0,5]	
Bajas temperaturas	Toda la jornada/mitad de la jornada	23 (76,7) [-2,5]	24 (48,0) [2,5]	0,012
	Ocasionalmente/nunca	7 (23,3) [2,5]	26 (52,0) [-2,5]	
Luz	Toda la jornada/mitad de la jornada	4 (13,3) [1,2]	12 (24,0) [-1,2]	0,240
	Ocasionalmente/nunca	25 (83,4) [-1,2]	36 (72,0) [1,2]	
Rayos de sol	Toda la jornada/mitad de la jornada	3 (10,0) [0,2]	6 (12,0) [-0,2]	0,816
	Ocasionalmente/nunca	25 (83,4) [-0,2]	42 (84) [0,2]	
Posturas incómodas	Toda la jornada/mitad de la jornada	16 (53,3) [-0,9]	21 (42,0) [0,9]	0,365
	Ocasionalmente/nunca	14 (46,7) [0,9]	28 (56,0) [-0,9]	
Cargar objetos pesados	Toda la jornada/mitad de la jornada	13 (43,3) [1,1]	28(56,0) [-1,1]	0,273
	Ocasionalmente/nunca	17 (56,7) [-1,1]	22 (44,0) [1,1]	
Sobre esforzar la voz	Toda la jornada/mitad de la jornada	5 (15,7) [0,0]	9 (18,0) [0,0]	0,987
	Ocasionalmente/nunca	22 (73,3) [0,0]	40 (70,0) [0,0]	
Trabaja sentado	Toda la jornada/mitad de la jornada	11 (36,7) [-1,7]	10 (20,0) [1,7]	0,092
	Ocasionalmente/nunca	18 (60,0) [1,7]	39 (78,0) [-1,7]	
Trabaja de pie	Toda la jornada/mitad de la jornada	26 (86,7) [1,1]	47 (94,0) [-1,1]	0,261
	Ocasionalmente/nunca	4 (13,3) [-1,1]	3 (6,0) [1,1]	
Uso de guantes	Sí	12 (40,0) [-0,9]	15 (30,0) [0,9]	0,360
	No	18 (60,0) [0,9]	35 (70,0) [-0,9]	
Uso de protector solar	Sí	2 (6,7) [-1,1]	1 (2,0) [1,1]	0,287
	No	28 (93,3) [1,1]	49 (98,0) [-1,1]	
Probabilidad de accidente	Muy probable	11 (36,7) [-0,4]	16 (32,0) [0,4]	0,894
	Poco probable	15 (50,0) [0,2]	26 (52,0) [-0,2]	
	Nada probable	4 (13,3) [0,3]	8 (16,0) [-0,3]	
La empresa se preocupa de su salud y seguridad	Siempre/casi siempre	9 (30,0) [1,5]	23 (46,0) [-1,5]	0,034
	Algunas veces	10 (33,3) [-2,5]	5 (10,0) [2,5]	
	Rara vez/nunca	11 (36,7) [0,5]	21 (42,0) [-0,5]	

Se observaron algunas diferencias por género respecto a la calidad de vida (Tabla 4), la variable “se sintió desanimado y triste” presentó diferencias significativas (p = 0,014), cuyos resultados fueron desfavorables para las mujeres, quienes en un 53,3% percibieron que casi todo el tiempo o un poco de este se ha sentido desanimada o triste, a diferencia de un 26% de la población masculina. Adicionalmente, cabe destacar que, en contraste al 12% de los hombres, un 33,3% de las mujeres calificaron la afirmación “siento que mi salud es excelente” como “casi siempre falso o definitivamente falso” (residuos tipificados corregidos: -2,3).

**Tabla 4:** Caracterización de estado de salud y calidad de vida de trabajadores de la Vega Central (Chile) por género.

Variables		Género		p

		Femenino Recuento (%) [Residuos corregidos]	Masculino Recuento (%) [Residuos corregidos]	
Dificultades para realizar trabajo/actividad	La mayor parte del tiempo/algunas veces	11 (36,7) [-0,2]	17 (34,0) [0,2]	0,809
	Pocas veces/nunca	19 (63,3) [0,2]	33 (66,0) [-0,2]	
Hacer menos de lo deseado por problemas emocionales	La mayor parte del tiempo/algunas veces	8 (26,7) [-1,0]	9 (18,0) [1,0]	0,318
	Pocas veces/nunca	21 (70,0) [1,0]	41 (82,0) [-1,0]	
Se sintió desanimado y triste	Casi todo el tiempo/un poco	16 (53,3) [-2,5]	13 (26,0) [2,5]	0,014
	Muy poco tiempo/nunca	14 (46,7) [2,5]	37 (74,0) [-2,5]	
Se sintió agotado	Siempre/casi todo el tiempo	25 (83,3) [-0.9]	36 (72) [0.9]	0,386
	Un poco/muy poco tiempo	5 (16,7) [-0.9]	12 (24) [-0.9]	
Siento que me enfermo con mayor facilidad	Definitivamente cierto/ casi siempre cierto	5 (16,7) [-1,9]	2 (4,0) [1,9]	0,057
	Casi siempre falso/definitivamente falso	24 (80,0) [1,9]	45 (90,0) [-1,9]	
Siento que estoy saludable como cualquiera	Definitivamente cierto/casi siempre cierto	22 (73,3) [1,3]	45 (90,0) [-1,3]	0,200
	Casi siempre falso/definitivamente falso	4 (13,3) [-1,3]	3 (6,0) [1,3]	
Siento que mi salud es excelente	Definitivamente cierto/casi siempre cierto	16 (53,3) [1,9]	37 (74,0) [-1,9]	0,065
	No sé	4 (13,3) [0,1]	7 (14,0) [-0,1]	
	Casi siempre falso/definitivamente falso	10 (33,3) [-2,3]	6 (12,0) [2,3]	

Respecto a la identificación de dicha autopercepción, se desarrollaron correlaciones respecto a las variables de calidad de vida, reconociendo asociaciones comunes en ambos géneros entre la autopercepción de salud y la limitación al caminar más de 10 cuadras (-0,417); limitación al subir solo un piso (-0,477); limitación de actividades por esfuerzos intensos (-0,412); limitación al caminar varias cuadras (-0,390); limitación al subir varios pisos (-0,567). Por otro lado, se identificaron correlaciones que solo eran de género femenino, tales como limitación a esfuerzos moderados y mínimos (-0,375 y -0,393, respectivamente), mientras que en hombres se obtuvieron niveles de correlación negativa significativas en las variables “siento que me enfermo con mayor facilidad” (-0,288); limitaciones en trabajo/actividad (-0,296); comparación de estado de salud con el año anterior (-0,299); reducción de tiempo de trabajo por problemas emocionales (-0,344); limitación al caminar solo una cuadra (-0,334); limitación al bañarse o vestirse (-0,354); se sintió desanimado y triste (-0,354); limitación al agacharse (-0,366) y limitación al levantar algo liviano (-0,369).

## DISCUSIÓN

En este estudio, se buscaba identificar diferencias de género en las condiciones de empleo, trabajo y calidad de vida de los/as trabajadores/as informales de la Vega Central. No se han identificado mayores diferencias de género en las condiciones de empleo y trabajo, pero las mujeres manifiestan un mayor temor que los hombres a reclamar mejores condiciones, así como se sienten más desanimadas y tristes durante la jornada.

Comparando con los datos nacionales de la misma encuesta utilizada como base (ENETS, 2011), llama la atención que a nivel nacional un 10,7% de los hombres y un 3,7% de las mujeres que son trabajadores/as informales no cuentan con sistema de previsión, mientras que en la Vega Central sobre un 60% de ambos géneros refiere no estar afiliado a un sistema previsional, aun cuando en el sistema público de salud exista la afiliación de personas sin ingresos formales. Por otro lado, un 20% de las mujeres trabajadoras de este mercado desconoce si tiene previsión de salud, demostrando mayor vulnerabilidad en este grupo.

Si bien la población femenina y masculina en el rubro estudiado no se sienten indefensos ante sus superiores, no presentan temor a ser despedidos/as, ni perciben tratos injustos o violentos, las mujeres manifiestan un mayor temor que los hombres a reclamar mejores condiciones laborales. Considerando que en la tendencia mundial del empleo informal se describe que las mujeres reciben una remuneración inferior por su trabajo y al mismo tiempo están expuestas a mayores déficits de trabajo decente^19^, es importante para este contexto implementar estrategias de seguridad social con perspectiva de género, que permitan a mujeres desarrollar herramientas y espacios para demandar justas condiciones.

La mayor parte de los/as participantes no cuenta con sistema previsional formal, no presentando brechas de género, lo que es contrario a lo reportado en otros estudios sobre trabajo informal^12^, en que la falta de seguridad social es mayor en mujeres que en hombres (4% frente a 1,5%). Esta falta de diferencia significativa puede atribuirse a que la población analizada se desempeña en un puesto de trabajo que no está feminizado o masculinizado, como sí podrían estar otros puestos de trabajo del ámbito informal. Debido al impacto en salud que esto conlleva, es un factor que se debe tomar en cuenta si se sabe que, en los países en desarrollo, el sector informal es la principal fuente de empleo para las mujeres^4^.

Si bien existe una heterogeneidad en la percepción, tanto de hombres como mujeres, de la preocupación del empleador sobre su salud y seguridad, se identifica una tendencia hacia la mayor percepción de negligencia por parte de las mujeres (p = 0,034). Podríamos vincular esta diferencia a aspectos culturales sobre la mayor relevancia que se da a la salud y la seguridad en el rol femenino. No obstante, dicha negligencia se expresa en los relatos similares de bajo acceso a capacitación, información y elementos de protección personal en su trabajo. A su vez, esto evidencia que en este rubro no existen prácticas claras respecto a las obligaciones del empleador a preocuparse del bienestar de trabajadores y trabajadoras, lo que es coincidente con otros estudios sobre trabajo informal^6,12,19^.

En general, solo factores físicos (posturas incómodas, carga de objetos pesados, jornadas trabajando de pie) presentan una exposición en más de la mitad de la muestra, mientras que se identificaron niveles de exposición global bajo un 30% a ruido, altas y bajas temperaturas o luz durante la jornada laboral. La única diferencia entre géneros en las condiciones de trabajo se identificó en el alto nivel de exposición a bajas temperaturas por parte de las mujeres (p = 0,012), lo cual podría atribuirse a que la mayor parte del día las mujeres refieren estar sentadas dedicadas a la venta en un espacio abierto y con techo alto que permite la circulación de aire como es el galpón donde se ubican los puestos de la Vega Central. En contraste, estudios similares tanto internacionales como nacionales han identificado que los hombres se exponen más a realizar trabajos en posturas incómodas^12^, bajo altas exigencias físicas y sufren más lesiones por accidentes de trabajo^20^, junto a mayores niveles de trabajo de pie, movimientos repetitivos, con levantamiento o traslado de objetos pesados, entre otros^14^. Pese a que la distribución de mujeres y hombres en el mercado laboral, en general, está dada por cómo se configuran las identidades femeninas y masculinas^21^, los resultados anteriores reflejan que en estos puestos de trabajo no existe una clara división de tareas ni de sus condiciones de trabajo por género, siendo ambos igualmente exigidos.

En relación a la calidad de vida, si bien la mayoría de los trabajadores se han sentido agotados la mayor parte del tiempo, no manifiestan dificultades para realizar su actividad laboral ni hacen menos de lo deseado por problemas emocionales. No obstante, las mujeres se perciben desanimadas y tristes durante la jornada en más de 2,01 (IC95%: 1,16–3,50) veces que los hombres, lo cual puede atribuirse a diversos factores, entre ellos, la diferente apertura a reconocer el estado anímico de hombres y mujeres, ya que de acuerdo al mandato hegemónico de la masculinidad un hombre no debe ser débil o reconocer tristeza. Por otro lado, este hallazgo es concordante con la estadística en la población general sobre salud mental, ya que mujeres presentan mayor prevalencia de síntomas depresivos que hombres^22^. Adicionalmente, la literatura sobre determinantes sociales de la salud y su impacto en la gradiente social de salud sitúa a los problemas y trastornos de salud mental con mayor prevalencia en grupos poblacionales con menor nivel educativo y menor edad^23,24^, elemento que considerar dado el bajo nivel educativo presentado en la muestra en general y en las mujeres en comparación a los hombres. Sin embargo, otro estudio sobre contexto de informalidad en América Central^11^ refiere que tanto hombres como mujeres presentan una mala percepción de salud mental, resultados similares a lo reportado en Chile, en que los trabajadores inestables e informales presentan significativamente menores niveles de bienestar mental respecto de los estables y formales^14^, por lo que el desánimo y la tristeza podrían estar igualmente asociados o bien potenciados por el trabajo informal y el bajo nivel educativo, que es a la vez una de las razones por las cuales se accede a este tipo de empleos.

Respecto a la autopercepción de salud en general, más del 60% señala como “definitivamente cierto/casi siempre cierto” que su salud es excelente, presentándose en un 53,3% de mujeres y un 74% de hombres. Esta similitud global en la salud autopercibida, si bien no es consistente con lo reportado en otros estudios^8,9,11^, puede ser explicada por la semejanza de las exigentes condiciones de trabajo y empleo en que ambos géneros desempeñan sus labores. A su vez, al describir las categorías “casi siempre falso/definitivamente falso”, se identificó una brecha de más del doble entre mujeres (33,3%) y hombres (12,2%), con un riesgo relativo de 2,07 (IC95% 1,18–3,62). Dicha asociación muestra mayor correlación en el caso de hombres a factores de origen físico y psicológico (niveles de correlación cercanos a -0,4), mientras que en mujeres muestra principal asociación con factores físicos en las tareas que realizan (con correlaciones significativas cercanas a 0,4). Otros estudios explican esta brecha por factores de riesgo físicos y sensoriales, doble presencia, baja calidad de liderazgo, bajo apoyo social y poca oportunidad de desarrollo de habilidades en el trabajo^26^ y conflictos de tiempo para conciliar demandas laborales y familiares en las mujeres^27^.

Con base en la literatura, otro de los factores que influyen en la autopercepción de salud de trabajadores informales corresponde a las características y experiencias de los sistemas de salud en los que participan las personas que trabajan en la Vega Central, tales como los problemas de administración de los servicios de salud, los malos tratos hacia los usuarios y la falta de recursos para el sector salud^28^. Además, Alfers et al.^8^ mostraron que en el contexto de informalidad laboral en Sudáfrica existe una fuerte asociación entre bajos ingresos y peor estado de salud, incluso mayor que con la formalidad laboral. En otras asociaciones con la salud, se ha visto que la exposición a riesgos psicosociales está mediada también por la clase social a la que la persona pertenece^28^.

El crecimiento del mercado de trabajo informal en América Latina se reconoce como una consecuencia del desarrollo del modelo económico, al cual se debe poner importante atención dada la tendencia a la informalidad como resultante de los procesos migratorios de la población hacia grandes ciudades^29^, considerando que la migración en Chile viene en aumento sostenido y que las políticas de salud y seguridad social siguen dejando fuera de sus reformas la situación de informalidad y los efectos en el bienestar de las trabajadoras y los trabajadores^30^.

Dentro de las limitaciones de este estudio, se encuentran las barreras presentadas en el proceso de reclutamiento de participantes y recolección de datos en el contexto del empleo informal, tanto por la carga temporal en el trabajo como por la extensión de los instrumentos. Si bien esto afecta la potencia estadística de los datos, se realizó un análisis post-hoc, encontrando valores superiores a 0,8 en las siguientes variables que presentan diferencias de género: “autopercepción de la salud”, “percepción de ánimo y temor a reclamar mejores condiciones”, y “preocupación de las empresas sobre su salud y seguridad en el trabajo”.

Otra limitación de este estudio es que dado su alcance descriptivo no profundiza en identificar las causas subyacentes a las diferencias de género en la percepción de salud, por lo que se requieren nuevos estudios que sean prospectivos o cualitativos, que puedan profundizar la comprensión del trabajo informal en mercados o ferias libres.

Los resultados de este estudio y su análisis permiten concluir que, a pesar de que en el rubro estudiado mujeres y hombres presentan similares condiciones de trabajo y laborales, las mujeres tienen peor percepción de su salud mental y salud general. Solo se identifican diferencias de género en la percepción sobre la preocupación del empleador por la salud y seguridad en el trabajo. Por lo tanto, las estrategias de promoción y prevención en salud laboral en los distintos rubros del trabajo informal, así como futuros estudios en este ámbito, deben diseñarse desde una perspectiva de género, incorporando tanto las condiciones laborales como los indicadores de salud y calidad de vida. Por otro lado, en cuanto avanzan las políticas de formalización del empleo y seguridad social, se pueden implementar estrategias alternativas para apoyar a madres y padres con el cuidado de hijos pequeños, tales como el desarrollo de convenios con servicios públicos locales para acceder a beneficios como el acceso a jardines infantiles, a los programas de extensión horaria de las escuelas, o bien contar con guarderías en el mismo recinto. Por lo anterior, toda medida e investigación en trabajo informal requieren considerar las particularidades de las mujeres que influyen en su desempeño y salud general en el trabajo.

## Footnotes

a Vegacentral.CL. Santiago (CL): Vega Central; c2020 [citado 1 Jun 2020]. Disponible en: https://www.vegacentral.cl/
